# Sporadic Vestibular Schwannoma Size and Location Do not Correlate With the Severity of Hearing Loss at Initial Presentation

**DOI:** 10.3389/fonc.2022.836504

**Published:** 2022-03-15

**Authors:** Alyssa Brown, Samuel Early, Sasa Vasilijic, Konstantina M. Stankovic

**Affiliations:** ^1^ Department of Otolaryngology—Head and Neck Surgery and Eaton-Peabody Laboratories, Massachusetts Eye and Ear, Boston, MA, United States; ^2^ Department of Otolaryngology—Head and Neck Surgery, Harvard Medical School, Boston, MA, United States; ^3^ Department of Otolaryngology Head and Neck Surgery, University of California, San Diego, San Diego Medical Center, San Diego, CA, United States; ^4^ Department of Otolaryngology—Head and Neck Surgery, Stanford University School of Medicine, Stanford, CA, United States

**Keywords:** vestibular schwannoma, magnetic resonance imaging (MRI), hearing loss, tumor size, tumor location

## Abstract

Vestibular schwannoma (VS) is a non-malignant intracranial neoplasm arising from the vestibular branch of the 8th cranial nerve; sensorineural hearing loss (SNHL) is the most common associated symptom. Understanding whether VS imaging characteristics at the time of VS diagnosis can be associated with severity of VS-induced SNHL can impact patient counseling and define promising areas for future research. Patients diagnosed with VS at Massachusetts Eye and Ear (MEE) from 1994 through 2018 were analyzed if magnetic resonance imaging at VS presentation and sequential audiometry were available. Results were compared with original studies available in PubMed, written in English, on VS imaging characteristics and their impact on hearing in patients. A total of 477 patients with unilateral VS from the MEE database demonstrated no significant correlation between any features of tumor imaging at the time of VS diagnosis, such as VS size, impaction or location, and any hearing loss metric. Twenty-three published studies on the impact of VS imaging characteristics on patient hearing met inclusion criteria, with six solely involving NF2 patients and three including both sporadic and NF2-related VS patients. Fifteen studies reported a significant relationship between SNHL and at least one VS imaging characteristic; however, these trends were universally limited to NF2 patients or involved small patient populations, and were not reproduced in larger studies. Taken together, SNHL in sporadic VS patients is not readily associated solely with any tumor imaging characteristics. This finding motivates future studies to define how VS microenvironment and secreted molecules influence VS-induced SNHL.

## Introduction

Vestibular schwannoma (VS) is the most common solitary, intracranial schwannoma, typically arising from Schwann cells of the vestibular branch of cranial nerve VIII. VS is also the most common cerebellopontine angle (CPA) tumor with a clinical incidence of about 1 per 100,000 persons (1, 2). Currently, observation *via* serial magnetic resonance imaging (MRI), surgical resection, and stereotactic radiation stand as the three management options for patients with VS (3–5). While asymmetric SNHL is the most common presenting symptom of patients with a VS (6, 7), the mechanisms of this SNHL remain incompletely understood.

VS-associated hearing loss was previously attributed solely to compression-induced compromise of the cochlear nerve or vascular supply to the cochlea; recent work calls attention to possible additional mechanisms, including VS-secreted ototoxic molecules, neuroinflammation, fibrosis, and edema within the tumor microenvironment (8). Improved MRI techniques have allowed for diagnosis of small VSs, highlighting the paradox of severe hearing loss occurring in some patients with an otherwise small VS, and normal hearing or only mild hearing loss in some patients with much larger tumors. Small VSs arguably pose the most complex determination of management, given their associated symptoms and size not warranting immediate intervention as clearly as for larger, compressive tumors. Utilizing high-resolution MRI protocols, protein deposition in the cochlea and labyrinthine hypo-intensity have been investigated as contributing factors to VS-associated hearing loss (9, 10). Further studies steer their focus towards the physical location of tumors, such as the superior vestibular nerve (SVN), and overall tumor size (11–14). The majority of such studies remain limited by small sample size, despite the availability and inclusion of strong audiometric and imaging data. Studies with larger populations struggle with inconsistent or incomplete MRI data (11, 15).

To provide deeper insight into mechanisms underlying VS-induced SNHL, we analyze the largest population of VS patients at a single institution with complete MRI and audiometric data at the time of VS diagnosis. In parallel, we present a comprehensive literature review of current, published papers related to tumor characteristics and hearing loss to better characterize trends identified across relevant studies. This combined analysis seeks to evaluate the degree to which any tumor imaging features can be associated with severity of hearing loss at the time of VS diagnosis, and to what extent these findings can be reliably applied across the diverse VS patient population.

## Materials and Methods

### Database Analysis

The charts of Mass Eye and Ear patients diagnosed with unilateral VS from January 1994 to October 2018 were previously identified and individually reviewed (16). Institutional Review Board (IRB) approval was obtained from the Mass General Brigham Human Studies Committee at Massachusetts Eye and Ear and Massachusetts General Hospital (IRB 16-103H). Audiometric data were analyzed from patient audiograms performed within 6 months of initial diagnosis based on contemporaneous MRI. Patients lacking a word recognition score (WRS) for the ipsilateral ear were included for analysis of study patient demographics, but excluded from analysis stratified by tumor size. Baseline WRSs of “pass” were replaced with a score of 92%. Word recognition testing was always performed at the sound level set for maximal performance, as evaluated using speech intelligibility index calculation. Linear and volumetric measurements were obtained on post-contrast T1-weighted MRI sequences within 6 months of initial diagnosis.

### Audiometric Data Calculations

For patients analyzed from the Mass Eye and Ear database, three separate pure-tone averages were calculated. Four-tone (speech-frequency) pure tone average (PTA) was calculated using thresholds at 0.5 kHz, 1 kHz, 2 kHz, and 4 kHz. High-frequency PTA was determined using thresholds at 3 kHz, 4 kHz, and 6 kHz. Low-frequency PTA was determined using thresholds as 0.5 kHz, 1 kHz, and 2 kHz (17, 18).

### Tumor Classification

**Figure 1:**
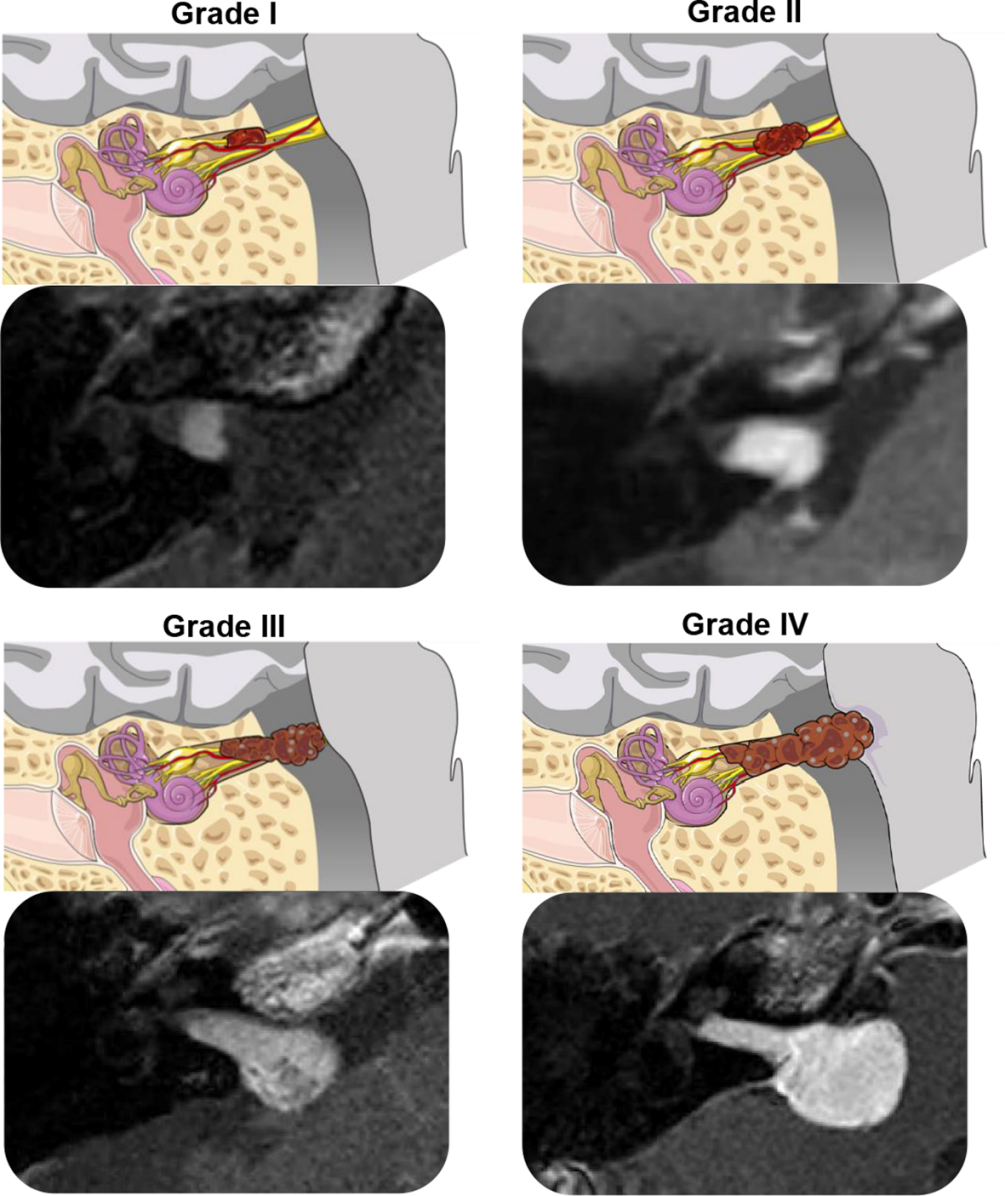
Illustrations and T1-weighted post-gadolinium MRI scans demonstrating the Koos Grading System for vestibular schwannoma (20). Grade I, small intracanalicular tumor; Grade II, small intracanalicular tumor with protrusion into the cerebellopontine angle; Grade III, tumor occupying the cerebellopontine cistern, touching but not compressing the brainstem; Grade IV, large tumor with brainstem compression. The illustrations were modified from SMART (Servier Medical Art), licensed under a Creative Common Attribution 3.0 Generic License (http://smart.servier.com).

For patients analyzed from the Mass Eye and Ear database, tumor size was assessed following the Koos grading system. Tumors were divided into four classes: Koos 1 (fully intracanalicular), Koos 2 (intracanalicular and extrameatal, but no brainstem contact), Koos 3 (touching the brainstem without compression), or Koos 4 (touching and compressing the brainstem) (19) (**Figure 1**). Tumor extent was organized into two categories, impacted and non-impacted. Impaction was defined as tumor extent to the fundus of the internal auditory canal (IAC), confirmed through visualization of solid enhancement and no cerebrospinal fluid signal at the cochlear fossette.

### Calculated Tumor Volume

For patients analyzed from the Mass Eye and Ear database, three-dimensional tumor volume was calculated as


$$Tumor\;volume=\frac{1}{6}\times \pi \times d1\times d2\times d3$$

where d1 and d2 are mediolateral and anteroposterior axial dimensions, respectively, and d3 is the largest craniocaudal dimension in a coronal plane (21, 22). Volumetric tumor analyses, although time-consuming, allows for more accurate and precise measurements (22–24). Calculated tumor volume allows for the closest approximation to using a semi-automated algorithm in determining tumor volume and saves the time it takes to manually segment an MR sequence (22). A widely accepted equation for calculating volume of VSs remains undetermined due to their asymmetry and lack of uniform edges (22, 24, 25).

### Maximum Linear Dimension

For patients analyzed from the Mass Eye and Ear database, maximum linear dimension (MLD) measurements were collected in the anteroposterior, mediolateral, and craniocaudal planes for each lesion. The MLD for each lesion was used for analysis. MLD is the current standard used in determining tumor size given the ease of measuring, but accuracy is comprised as the other two linear measurements are not reported. This has the potential to lead to under- and overestimations with reduced sensitivity in determining tumor growth when compared to volumetric measurements (22, 24, 26–28).

### Cross-Sectional Area

For patients analyzed from the Mass Eye and Ear database, cross-sectional area was determined following the Macdonald criteria equation


Tumor area=d1 × d2

where d1 is the MLD in the mediolateral plane and d2 is the maximum dimension perpendicular to d1 (29). Given the use of two linear dimensions, the cross-sectional area of tumors is more in-line with calculated tumor volume and is often the chosen method of tumor size reporting.

### Literature Review

**Figure 2:**
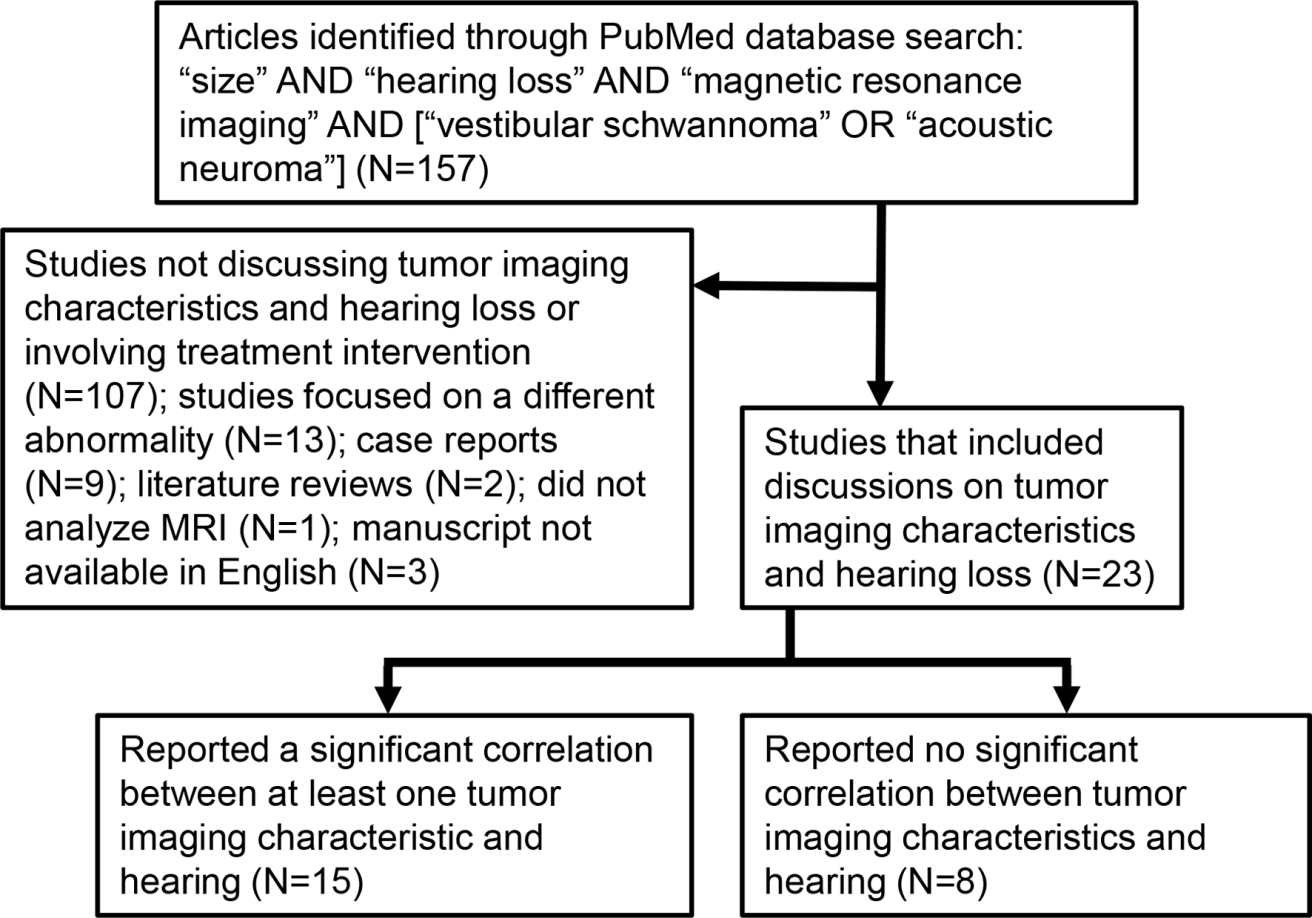
Selection of articles through comprehensive literature review.

Peer-reviewed papers available on PubMed, published prior to September 2021 and written in English on the topics of VS, MRI, tumor size, growth, and/or location, and hearing loss were identified as schematized in **Figure 2**. An article had to involve a study of VS-associated hearing loss through MRI analyses. The initial search identified a total of 157 papers; 23 of these met our study criteria because they focused on VSs prior to an intervention and discussed at least one of tumor size, location, growth rate, or MRI characteristics of the inner ear in relation to VS-associated SNHL.

### Statistical Analysis

GraphPad Prism version 8.0 for Windows (GraphPad Software, San Diego, CA, USA) was used for data analyses. One-way analyses of variance (ANOVAs) were used to compare groups. The coefficients of determination (*R* ^2^) were calculated for correlation analysis. A *p*-value of <0.05 was determined to be significant.

## Results

### Characteristics of Study Patients From Mass Eye and Ear Database

**Figure 3:**
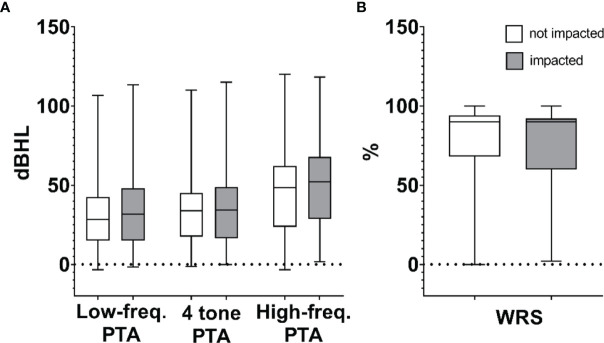
Composite box plots demonstrating patient audiometric data for non-impacted and impacted tumors calculated as **(A)** high-frequency pure tone average (PTA), speech-frequency (4-tone) PTA, low-frequency PTA, and **(B)** word recognition score (WRS). Within each box, horizontal lines denote median values; boxes extend from the 25^th^ to the 75^th^ percentile of each group’s distribution of values; vertical extending lines denote adjacent values (i.e., the most extreme values within 1.5 interquartile range of the 25^th^ and 75^th^ percentile of each group).

The demographics, tumor classification, and audiometric data of patients included in the study are shown in **Table 1**. The average age was 55.5 years, with 269 female (56.4%) and 208 male (43.6%) patients. Koos classifications 1–4 comprised 188 (39.4%), 144 (30.2%), 24 (5.0%), and 121 (25.4%) patients, respectively. Calculated mean PTAs in the ears ipsilateral to VS at the time of initial diagnosis were 34 dB, 47 dB, and 31 dB at speech-range, high, and low frequencies, respectively. Average ipsilateral WRS at the time of initial diagnosis was 77%. Differences in hearing levels (evaluated by PTA and WRS) between impacted and non-impacted tumor subgroups were not statistically significant (*p* > 0.05), as summarized in box plots (**Figure 3**).

**Table 1:** Patient demographic data.

Sex, *n* (%)	
Male	208 (43.6%)
Female	269 (56.4%)
Average age, year (95% CI)	55.5 (54.4, 56.6)
Male	56.2 (54.5, 57.8)
Female	54.9 (53.4, 56.5)
Mean tumor volume, cm^3^ (SD), *n*	1.84 (4.04), 472
Patients in Koos class 1, *n*	0.09 (0.27), 186
Patients in Koos class 2, *n*	0.54 (0.69), 142
Patients in Koos class 3, *n*	1.42 (0.95), 24
Patients in Koos class 4, *n*	6.09 (6.24), 120
Initial speech-frequency pure-tone average, dB (SD)	34 (19)
Initial high-frequency pure-tone average, dB (SD)	47 (25)
Initial low-frequency pure-tone average, dB (SD)	31 (20)
Initial word recognition score, % (SD)	77 (28)

Patients without complete or available MRI to allow for impaction analysis at the time of diagnosis were excluded from the base set, yielding 477 included patients. Tumor size analysis by tumor volume calculation yielded 472 patients as coronal cuts on MRI were unavailable for five patients. Baseline WRS for the ipsilateral ear was not available for 16 of the 477 patients. Baseline WRSs of “pass” replaced with scores between 92% and 96% resulted in directionally similar findings (*R* ^2^ ≤ 0.06 regardless of method used to handle “pass” results).

**Figure 4:**
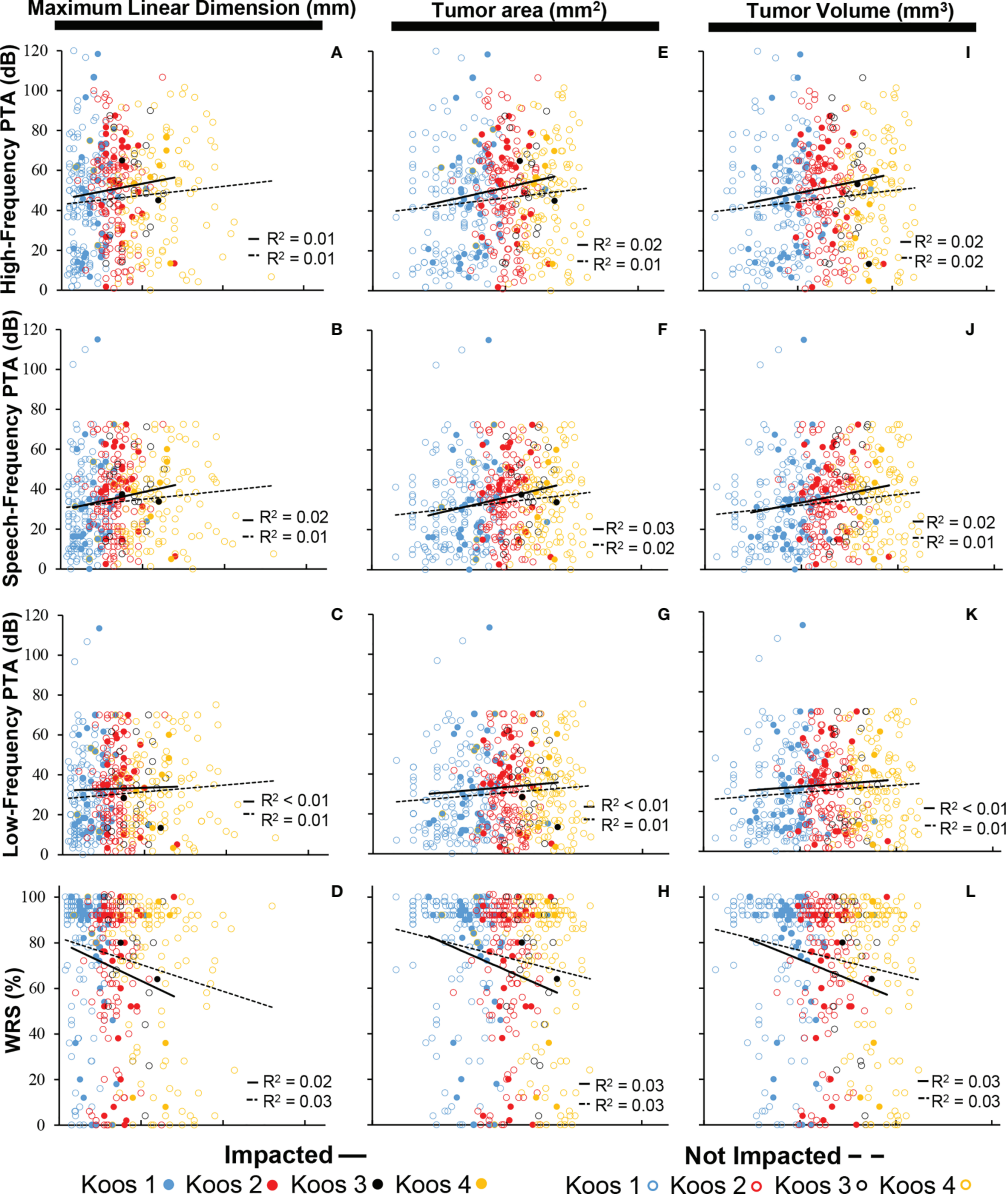
Composite scatter plots showing the relationship in impacted and non-impacted groups between maximum linear dimension and PTA (*N* = 477) calculated as **(A)** high-frequency PTA, **(B)** speech-frequency PTA, and **(C)** low-frequency PTA; **(D)** maximum linear dimension and WRS (*N* = 461); cross-sectional area and PTA (*N* = 477) calculated as **(E)** high-frequency PTA, **(F)** speech-frequency PTA, and **(G)** low-frequency PTA; **(H)** cross-sectional area and WRS (*N* = 461); tumor volume and PTA (*N* = 472) calculated as **(I)** high-frequency PTA, **(J)** speech-frequency PTA, and **(K)** low-frequency PTA; and **(L)** tumor volume and WRS (*N* = 461). PTAs calculated in all three frequency ranges are not significantly associated with tumor size within both the impacted and non-impacted groups (*p* > 0.05). WRS is not significantly associated with tumor size within both the impacted and non-impacted groups (*p* > 0.05). PTA, pure tone average; WRS, word recognition score.

**Figure 4** shows that no significant correlation exists between tumor size and hearing, regardless of the specific metric used for tumor size (measured as MLD, tumor cross-sectional area, or tumor volume) or for hearing (high-, speech-, or low-frequency PTA, or WRS). No correlations of significance were found regardless of whether or not tumors were impacted (*R*² ≤ 0.03 in all cases). Furthermore, multiple comparison ANOVAs did not demonstrate significance between groups stratified by Koos classification. Data in **Figure 3** are replotted focusing on MLD (**Supplementary Figure 1**), tumor cross-sectional area (**Supplementary Figure 2**), or tumor volume (**Supplementary Figure 3**) with different colors indicating different Koos groups.

### Key Findings From Literature Review

Twenty-three studies reporting on tumor imaging characteristics (MRI signal, size, location, impaction, or growth) and hearing were found, with fifteen demonstrating at least one significant, direct correlation between at least one tumor imaging characteristic and hearing ability (**Figure 2**). **Table 2** shows that the most common MRI sequence used for analysis was post-gadolinium T1-weighted sequence, with fourteen studies including only patients with sporadic VS, six studies with only Neurofibromatosis type 2 (NF2) patients, and three including both patient groups. Study sample sizes ranged from 7 to 534 patients, with a total population of 2435 VS patients. Eight studies involved investigation of the internal auditory canal extent of tumors or fundal cap size, with only two of these studies referencing obstruction (tumor impaction) of the cochlear aperture.

**Table 2:** Summary of historical analyses of hearing loss vs. imaging characteristics in patients with vestibular schwannoma.

Reference	Size Technique	*N*	Imaging Sequence	Impaction	Tumor Characteristics	Outcome	
						Significant	Not significant
**Berrettini et al.** (30)	MLD	42	MRI+ T1	IAC extent	Sporadic, unilateral Evaluation at initial presentation	–	No significant difference of HL between different sized tumors. No significant correlation of tumor origin and subjective HL.
**Nadol et al.** (31)	MLD IAC extent	75	MRI+ T1	IAC extent	Sporadic, unilateral Preoperative evaluation	Significant correlation between MLD and low-frequency SNHL severity.	No significant correlations between MLD and SNHL severity in high/mid-frequencies or SDSs, and lateral extent of the tumor within the IAC and SDSs.
**Lalwani et al.** (32)	Tumor volume Linear dimensions	40	MRI	–	NF2 Evaluation at initial presentation	PTA significantly worse in the “larger tumor” groups. Worsening SRT with larger tumor size (TV dimension, volume).	PTAs for individual frequencies were not correlated with tumor size.
**Tanaka et al.** (33)	MLD	34	MRI	–	Sporadic, unilateral Diagnostic intervention	–	No significant correlation between the detective threshold of compound action potential or cochlear microphonics (ECochG) and tumor size.
**Massick et al.** (34)	MLD Tumor volume	21	MRI+ T1	–	Sporadic, unilateral NF2 Sequential follow-up	Increasing TV correlates with HL (increased PTA, decreased WRS). Decline of initial auditory function class corresponds with an even quicker rate of audiometric decline with tumor growth.	–
**Tschudi et al.** (35)	MLD	74	MRI	–	Sporadic, unilateral Evaluation at initial presentation Sequential follow-up	–	Higher-frequency thresholds were more impacted than lower frequencies, but no significant correlation between tumor size and initial HL. No significant correlation between tumor growth and HL.
**Wang et al.** (36)	MLD	7	MRI	–	NF2 Diagnostic intervention	–	No significant relationship between tumor size and hearing level.
**Caye-Thomasen et al.** (15)	Tumor volume Localization	156	MRI- T2	IAC extent	Sporadic, unilateral Intracanalicular Evaluation at initial presentation Sequential follow-up	Significant correlation between absolute volumetric tumor growth rate and PTA deterioration rate.	HL diagnosis at time of presentation is irrespective of patient demographics, tumor sublocalization, and tumor-induced expansion of the IAC.
**Day et al.** (37)	MLD	44	MRI T1 MRI T2 MRI Proton density	IAC extent	Sporadic, unilateral Diagnostic intervention	Significant trend of correlation with tumor size and HL.	–
**Fisher et al.** (38)	MLD	52	MRI+ T1 MRI T2	–	NF2 Sequential follow-up	–	No significant association between VS size hearing for either side. No significant relation between change in tumor size and hearing deterioration.
**Gerganov et al.** (39)	MLD Tumor volume	99	MRI- T1 MRI- T2 MRI+ T1 MRI+ T2	IAC extent Tumor-fundus distance	Sporadic, unilateral Preoperative evaluation	Hearing ability correlated significantly with the tumor size, volume and coronal diameter, the degree of intrameatal tumor growth, and the distance between tumor end and fundus.	–
**Sughrue et al.** (40)	Localization	59	MRI	–	Sporadic, unilateral NF2, unilateral Evaluation at initial presentation Sequential follow-up	Hearing is lost at a quicker rate in faster-growing tumors than slow-growing tumors.	Initial tumor size at diagnosis did not significantly affect the time to serviceable HL.
**Van de Langenberg et al.** (12)	Tumor volume	63	MRI+ T1 MRI- T2	–	Sporadic Evaluation at initial presentation Sequential follow-up	Labyrinthine hypo-intensity (T2) and HL complaints at presentation predictive of faster hearing decline.	TV and change in TV does not correlate significantly with HL.
**Asthagiri et al.** (41)	Tumor volume	56	MRI- T1 MRI- FLAIR MRI+ T1 Co-registered T2-VISTA MRI+ delayed FLAIR	Cochlear aperture obstruction	NF2 Diagnostic intervention	–	Association between HL and tumor size (TV) is not strong enough. HL appears to possibly develop from cochlear aperture obstruction and intralabyrinthine protein accumulation.
**Tutar et al.** (42)	MLD (extrameatal)	76	MRI	–	Sporadic, unilateral Preoperative evaluation	–	No correlation found between tumor size and hearing levels at each frequency.
**Holliday et al.** (9)	Tumor volume	32	MRI+ T1 MRI- T2 TSE VISTA MRI- FLAIR	Cochlear aperture obstruction	NF2 Diagnostic intervention	Elevated intralabyrinthine protein correlated with larger tumors. Significant association between aperture obstruction and 4-tone PTA and ABR changes.	Tumor volume was not significantly correlated with 4-tone PTA.
**Plotkin et al.** (43)	Tumor volume	120	MRI+ T1	–	NF2 Sequential follow-up	Significant difference in time to hearing decline with medium/large tumors having a shorter median time to hearing decline compared with small tumors.	–
**Van Linge et al.** (44)	Tokyo consensus Localization	155	MRI+ T1 MRI- T2 FIESTA MRI- T2 CISS	–	Sporadic, unilateral Sequential follow-up	Tumor growth associated with faster AHDR for intracanalicular tumors.	PTA or SDS in the ipsilateral ear did not differ between classes of intensity of the cochlear fluid signal.
**Kirchmann et al.** (13)	MLD Localization	156	MRI	–	Sporadic, unilateral Sequential follow-up	Hearing is lost at a significantly faster rate in growing tumors.	Rate of SDS decrease is not significantly associated with tumor growth. No significant difference between HL progression in patients with intrameatal versus extrameatal tumors.
**West et al.** (45)	MLD (extrameatal)	124	MRI	–	Sporadic, unilateral Extrameatal Preoperative evaluation	–	Caloric tests and VEMPs are potential clinical factors for measuring tumor size, sensitive but remain unspecific. No correlation between increasing tumor size and HL and peripheral vestibular function.
**Byun et al.** (14)	MLD Localization	23	MRI+ T1 MRI- T2	–	Sporadic, unilateral Evaluation at initial presentation	Intracanalicular tumors associated with increased DRs than extracanalicular tumors.	No strong correlation between tumor size and WRS/PTA. No significant correlation with PTA and T2-weighted signal intensity.
**Early et al.** (16)	MLD	534	MRI	–	Sporadic, unilateral NF2 Evaluation at initial presentation Sequential follow-up	Patients with abnormal baseline hearing of the ipsilateral ear, demonstrated significantly higher likelihood of reaching moderate SNHL in the contralateral ear.	Patients with normal baseline hearing bilaterally demonstrated no significant difference in HL progression in VS-contralateral vs. control ears. Subgroup analysis by baseline tumor size did not show any specific trends for HL progression.
**Selleck et al.** (11)	MLD Cochlear FLAIR ratio	393	MRI- T2 FLAIR MRI- T2 CISS	Fundal cap size	Sporadic, unilateral Evaluation at initial presentation	An indirect, significant relationship exists between initial WRS and cochlear FLAIR ratio. Significant correlation was seen between decreasing WRSs and increasing fundal cap size.	No statistically significant correlation between initial PTA and cochlear FLAIR ratio. No statistically significant correlation between initial WRS and PTA, and fundal cap.

Of the papers demonstrating a direct relationship between tumor characteristics and hearing loss, four drew conclusions based solely on tumor size, while the remaining investigated multiple tumor characteristics, such as size, growth, aperture encroachment and involvement, and MRI signal intensity. From papers demonstrating at least one significant relationship, patient sample size ranged from 21 to 534 patients, with an average of 131 patients. Three studies included NF2 patients, nine included patients with sporadic, unilateral tumors, and three included both sporadic VS and NF2-associated tumors (**Table 2**).

### Tumor Size vs. Hearing Loss at Initial Presentation

Two studies demonstrated a weak trend towards loss of higher frequency thresholds with increasing tumor size; however, no significant correlation was seen across all frequencies in patients with sporadic tumors (35, 42). When hearing was assessed using electrocochleography, there was no direct impact of tumor size on the detective thresholds of compound action potential or cochlear microphonics (33). A group of studies using combined PTAs and speech discrimination score (SDS) to represent hearing drew no significant relationship between tumor size and hearing loss in the tumor-ipsilateral ear for patients with sporadic tumors (12, 14, 30, 40, 45). Higher PTAs and lower WRSs in patients with larger tumors were demonstrated separately in NF2 cohorts, however (32, 43). Contributions by Lalwani et al. confer audiologic profile predictability to the severity of NF2 disease, with milder NF2 cases showcasing predictable audiologic profiles vs. patients with clinically severe NF2, while Plotkin et al. suggest a stratification approach to determine a patient’s risk for subsequent hearing decline based purely on tumor volume—again, strictly for patients with NF2 disease (32, 43).

### Tumor Impaction and Localization as Predictors of Hearing Loss

Evaluation of impaction and localization across studies did not consistently show significant correlation with hearing loss (30). Localization becomes a debated factor with different patient groups demonstrating conflicting results. Studies showing a significant correlation of localization and hearing found both increased annual hearing decline rate (AHDR) and increased cochlear dead regions (DRs) in intracanalicular tumors when compared to those mainly within the CPA, which potentially supports the mechanistic theory of increased mechanical compression of the nerve within the IAC of intracanalicular tumors (14, 44). In one study, the distance between the lateral-most portion of tumor and the fundus had a direct correlation with worse hearing (39). An even greater number of studies, however, show no significant difference in patients’ hearing between fundus, central, or porus-centered tumors (13, 15). Furthermore, no correlation is found between lateral extent within the IAC and hearing ability, precluding a strict sublocalization criteria as a determining factor for tumor intervention (11, 15, 31).

Direct tumor impaction at the cochlear fossette was reported in two studies, but the study populations comprised only NF2 patients (9, 41). Impaction investigated as the simple association of tumor size and hearing loss has not been robust enough to explain fully the timing of hearing loss onset and progression in NF2 patients (41). Separately, cochlear aperture obstruction is believed to play a role as worsening 4-tone PTAs and auditory brainstem evoked responses were significantly associated with such impacted tumors, relying again upon the argument of pressure applied to the cochlear nerve by tumor-displaced cerebrospinal fluid towards the IAC apex (9). Such a mechanism for increased pressure is not supported when evaluating expansion of the IAC and patients’ hearing in sporadic, unilateral tumors, however (15).

### Tumor Imaging Characteristics vs. Hearing Loss Progression

Beyond hearing at initial presentation, hearing loss progression has also been a common topic of study for clinical prognostication of anticipated hearing loss over time. The earliest study looking at the effect of tumor growth over time does show decreased WRSs and increased PTAs in both NF2 and sporadic VS patients (34). A similar result is reported in later literature, with the correlation only holding for patients with intracanalicular tumors, thus providing a modicum of support to the theory of nerve compression as the sole cause for worsening hearing loss (44). A lack of proportionality, however, was witnessed between growing tumors and hearing function, which undermines generalizability of results across patients (40). Multiple studies illuminate the possibility of interplay between multiple tumor characteristics and disease factors, but with no single discrete imaging feature explaining a dominant fraction of total variance. Anatomical compression of the cochlear nerve is reliably demonstrated to have no significant correlation with hearing progression across multiple studies (12, 15, 35).

## Discussion

This study, to our knowledge, represents the most comprehensive assessment of the relationship between hearing function and tumor MRI characteristics to date in patients presenting with VS, based not only on our own institution-specific patient population but also on compiled meta-analysis. Our database for sporadic VS patients augments the literature by providing the largest patient population to date (representing 22% of all patients analyzed in this study) in which reinterpretation of the primary data was feasible (16). Analyzing by an exhaustive range of tumor size factors including maximal linear dimension, cross-sectional area, total volume, impaction, and Koos classification all yielded no correlation with hearing loss at the time of VS diagnosis. These findings are in agreement with broader findings across published literature, and ultimately reject mechanical pressure of the vestibulocochlear nerve as the only factor determining hearing ability (12, 15, 31, 35, 38).

Tumor size stands as the most prominent independent factor studied across related literature looking at baseline hearing, or when performing diagnostic analyses at a given time point. A broad range of papers have consistently shown no significant correlation between tumor size and hearing loss at time of sporadic VS diagnosis, introducing other possible factors for influencing hearing at time of initial presentation (12, 14, 30, 33, 35, 40, 42, 45). In the only study to demonstrate any correlation between tumor size and risk of hearing loss progression in sporadic tumors, results may not be generalizable given the small patient population (*N* = 21); furthermore, the most significant correlations found between tumor size and hearing loss progression were only present in patients with AAO-HNS Class D hearing at baseline, defined as WRS less than 50%, severely restricting clinical utility of this finding (34). Heterogeneity in hearing loss progression, regardless of the rate at which the tumor does or does not grow, also complicates the ability to assign a generalizable relationship between tumor growth and hearing changes (40).

Some studies have found an occasional, constrained correlation between other VS imaging characteristics and hearing at the time of initial presentation. Following the premise of IAC pressure and nerve compression, patients with larger tumors demonstrated greater hearing deterioration than those with smaller tumors (34, 37). In parallel to compression neuropathy, acute ischemic events, and overall restriction of adjacent vascular structures are offered as explanations for hearing loss in patients with larger tumors and are supported by significant correlative data (31, 34, 37). Decreased hearing ability is also attributed to larger tumors due to possible nerve fiber stretching and further vascular compromise of the cochlea (31, 39). Both biochemical and biophysical interference with cochlear function are described in sporadic VS patients, and this extends to NF2 patient cohorts (32, 43). Similar to sporadic VS studies, those focusing on NF2 populations also highlighted the lack of a clear size threshold for symptom severity, and the need for more complex clinical studies to tackle discordant results between tumor size and hearing in NF2 patients (9, 36, 38, 41).

Indirect, secondary factors are then pushed to the forefront, such as consideration of intratumoral inflammation, hemorrhage, fibrosis, ischemic necrosis, and alterations in the biochemical composition of the inner ear, to account for sporadic VS-related hearing deterioration (12, 15, 35, 38). Even within a single NF2 patient cohort, a simple genotype–phenotype relationship is not representative of the disease and patient presentations, thus increasing suspicion for vascularity as an important culprit (38, 40). Hearing loss progression in patients with a slow-growing or non-growing tumor supports investigation into the vascular impacts within the IAC and inner ear, because it has been suggested that a highly vascularized tumor may “steal” blood from the nearby cochlea (12, 35). In parallel, quantitative data show increased expression of vascular endothelial growth factor, as well as overall strengthening of the interaction that a growing tumor will have on surrounding vascularity and inflammation (38, 46–49).

Interestingly, increased intralabyrinthine protein deposition has previously been observed and correlated with tumors encroaching the cochlear fossette (9, 12, 41). Signal significance was observed in a portion of these studies, both during baseline analysis and throughout observation (9, 12). Although changes in intralabyrinthine fluid signal are suggestive of increased protein in the CSF and perilymph due to tumor presence, the link to either hearing loss or hearing loss progression has not met criterion for significance (11, 14, 41). Protein deposition, specifically in the scala media, has recently provided a correlative impact on hearing loss in VS as well (8, 9). The mechanism of such deposition, seen on fluid-attenuated inversion recovery MRI sequences, remains an active area of study (9, 41). Parallel studies showcase the abundance of tumor-secreted factors, such as matrix metalloproteinase-14 (MMP-14), capable of causing SNHL through spiral ganglion neuron fiber and synapse damage (8). Additional molecular biomarkers identified throughout the inflammatory microenvironment of VS that may contribute toward tumor-induced hearing loss include tumor necrosis factor alpha (TNFα), IL-6, CXCR4, and nuclear factor kappa-B (NFκB) activation (50–53).

Intralabyrinthine MRI signal, although suggestive of tumor-induced inflammation and consequent hearing loss progression, has also fallen short of significance (44). Studies investigating the role of T2-weighted hypo-intensity on MRI, protein deposition, and secreted factors in VS patients offer an avenue for additional research to understand biochemical alterations occurring within the IAC and inner ear when a tumor is present, and as possible factors contributing to hearing loss (54).

The systematic literature review has several limitations. The geographical distribution of article sources is restricted primarily to studies in North America and Western Europe, risking introduction of ethnicity bias. The majority of reviewed papers studied small populations. Moreover, impacted tumors are infrequently encountered in clinic, rendering this study subgroup much smaller compared to the more common non-impacted tumors. Other confounding factors, such as age and retrocochlear function, which are known contributors to hearing loss, were not analyzed in this study. Incomplete records or unavailability of imaging and audiometric data limit all retrospective studies. Additionally, some patients who frequented Massachusetts Eye and Ear for VS-related follow up had their initial MRI performed at an outside facility, possibly affecting measurement accuracy depending on the MRI protocols utilized across various facilities. An additional limitation is that tumor volume was calculated by multiplying three linear dimensions, not by an algorithm that requires tracing of tumor scans. Overall, caution should be taken in applying conclusions based on NF2 populations more broadly to the greater population of sporadic unilateral VS patients—in NF2 patients, the correlation between tumor size and growth with hearing loss is more easily demonstrated; however, in general, these tumors are typically more rapidly growing than sporadic VS, and more histologically aggressive, with NF2-associated tumors more frequently directly involving the cochlear nerve (55, 56).

The need to better characterize factors contributing to the complexity of hearing loss in patients with VS continues to be highlighted by the presented results, since readily measured imaging variables such as tumor size, location, and growth rate do not individually account for the observed tumor-associated SNHL at the time of presentation (15, 31, 54). More advanced imaging or other diagnostic studies, regarding evidence of protein deposition *via* fluid-attenuated inversion recovery MRI sequences, combined with measuring the cochlear inflammatory microenvironment *via* markers such as TNFα, IL-6, CXCR4, MMP-14, and NFκB activation, may yet provide insight into which tumors are at higher or lower risk of affecting hearing. Quantification of circulating molecular biomarkers may also inform perspective on surgical candidacy between several patients with otherwise equivalent baseline hearing and other tumor imaging characteristics. Further identification of VS circulating biomarkers would be critical for future clinical trials, not only holding the potential for detecting earlier tumor growth and risk of SNHL, but also serving as therapeutic targets.

## Data Availability Statement

The datasets presented in this article are not readily available because the data analyzed in this study are subject to the following licenses/restrictions: Datasets can be made available upon request to interested parties. The primary dataset includes service dates for certain procedures that are considered protected under HIPAA guidelines, and as such cannot be shared publicly. Requests to access the datasets should be directed to kstankovic@stanford.edu.

## Ethics Statement

The studies involving human participants were reviewed and approved by Mass General Brigham Institutional Review Boards (IRB). Written informed consent for participation was not required for this study in accordance with the national legislation and the institutional requirements.

## Author Contributions

KS conceived the project, analyzed data, and supervised all aspects of the research, including critical editing of the manuscript. SE performed initial patient chart reviews and created the VS patient database. AB performed MRI analyses for tumor impaction and completed literature review. AB, SE, and SV analyzed the data. AB and SE wrote the manuscript. All authors critically edited the manuscript. All authors contributed to the article and approved the submitted version.

## Funding

This work was supported by NIDCD grant R01 DC015824, Larry Bowman, and the Remondi Foundation (KS). KS gratefully acknowledges support from the Bertarelli Foundation Professorship.

## Conflict of Interest

The authors declare that the research was conducted in the absence of any commercial or financial relationships that could be construed as a potential conflict of interest.

## Publisher’s Note

All claims expressed in this article are solely those of the authors and do not necessarily represent those of their affiliated organizations, or those of the publisher, the editors and the reviewers. Any product that may be evaluated in this article, or claim that may be made by its manufacturer, is not guaranteed or endorsed by the publisher.
