# Supplementary figures and images for: Sporadic Vestibular Schwannoma Size and Location Do not Correlate With the Severity of Hearing Loss at Initial Presentation
Source: Front Oncol. 2022 Mar 15;12:836504. doi: 10.3389/fonc.2022.836504 (PMC8965062; doi:10.3389/fonc.2022.836504)

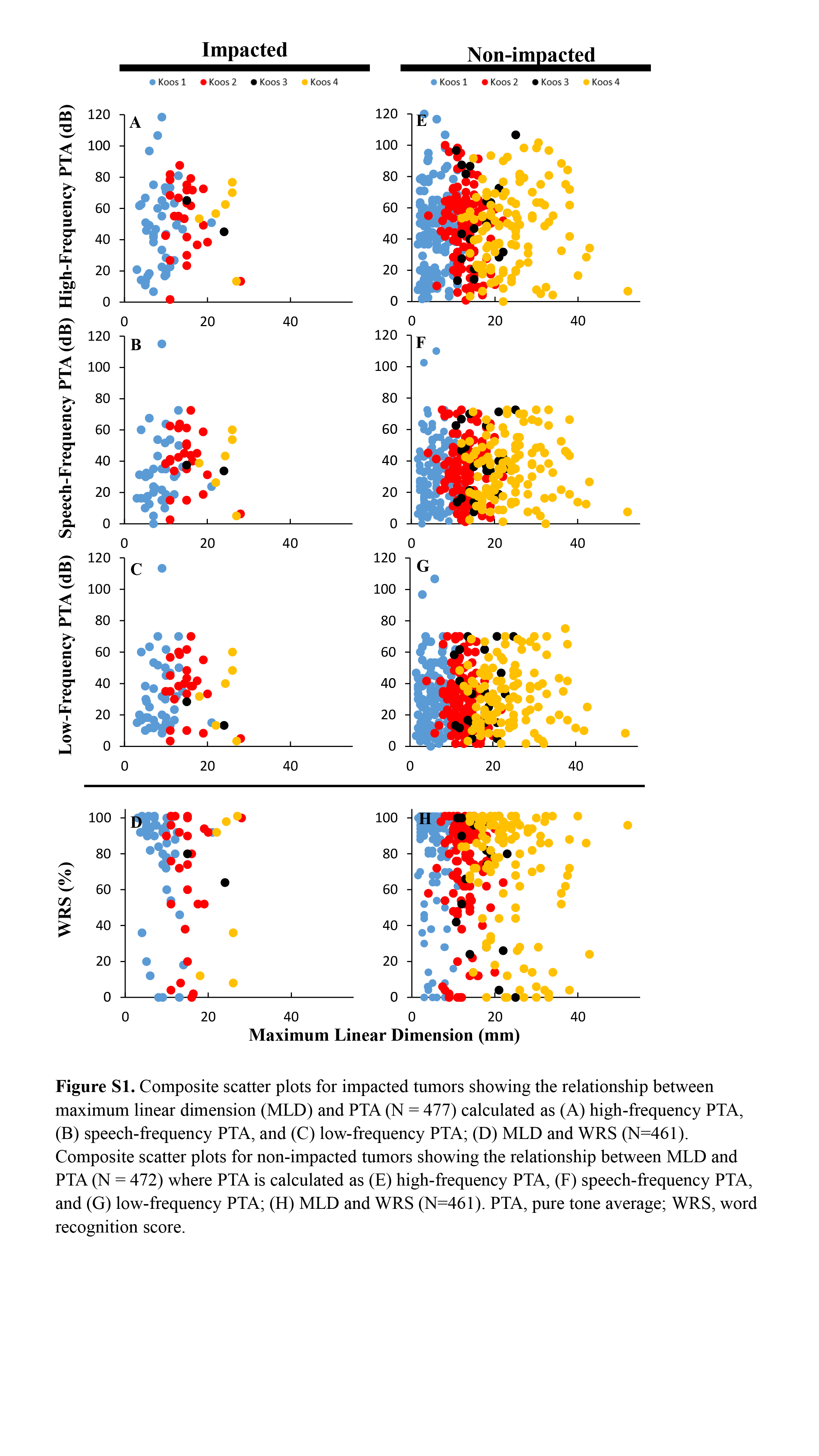

Supplement: Supplementary file 1 [file Image_1.tif]

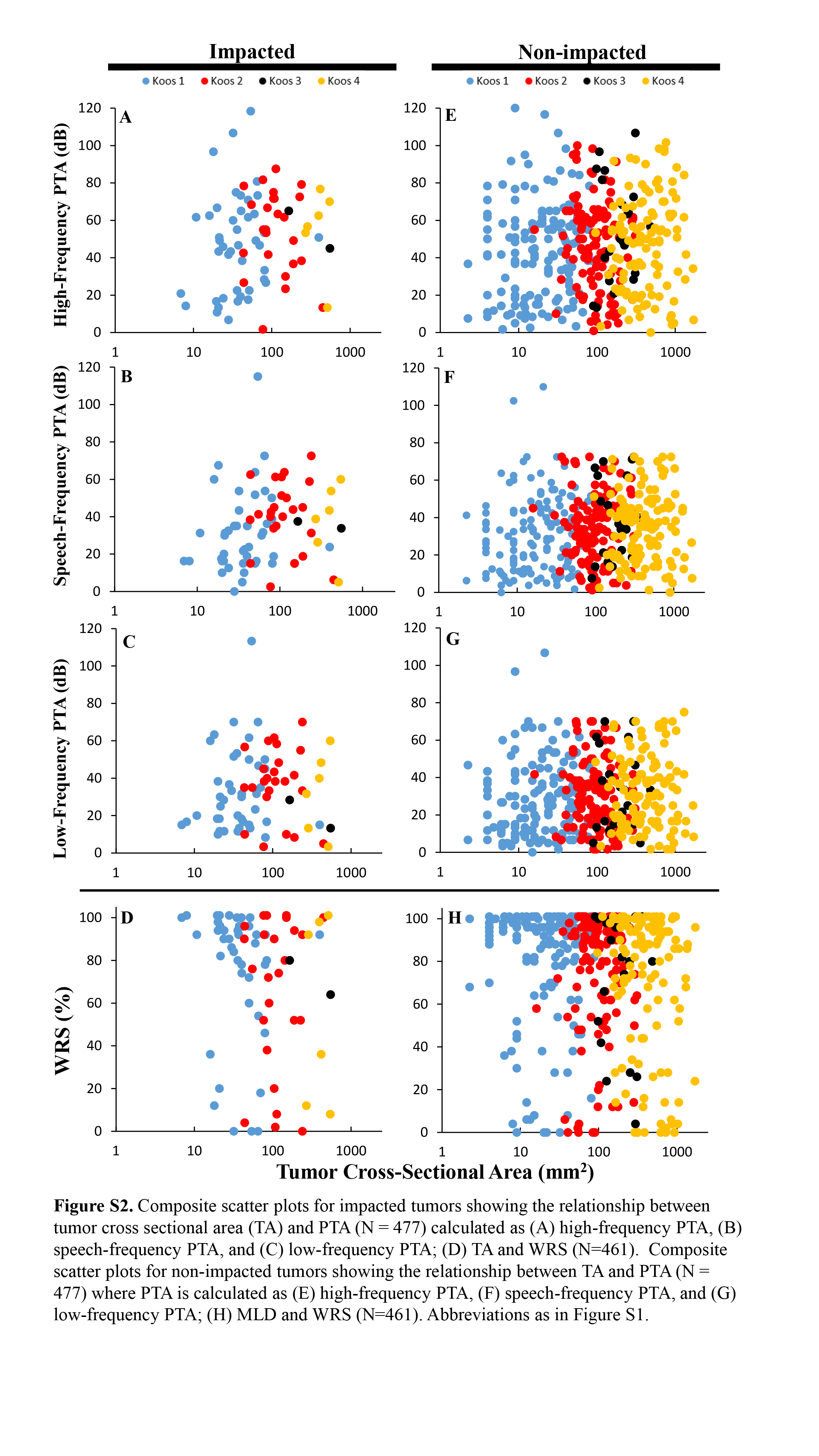

Supplement: Supplementary file 2 [file Image_2.tif]

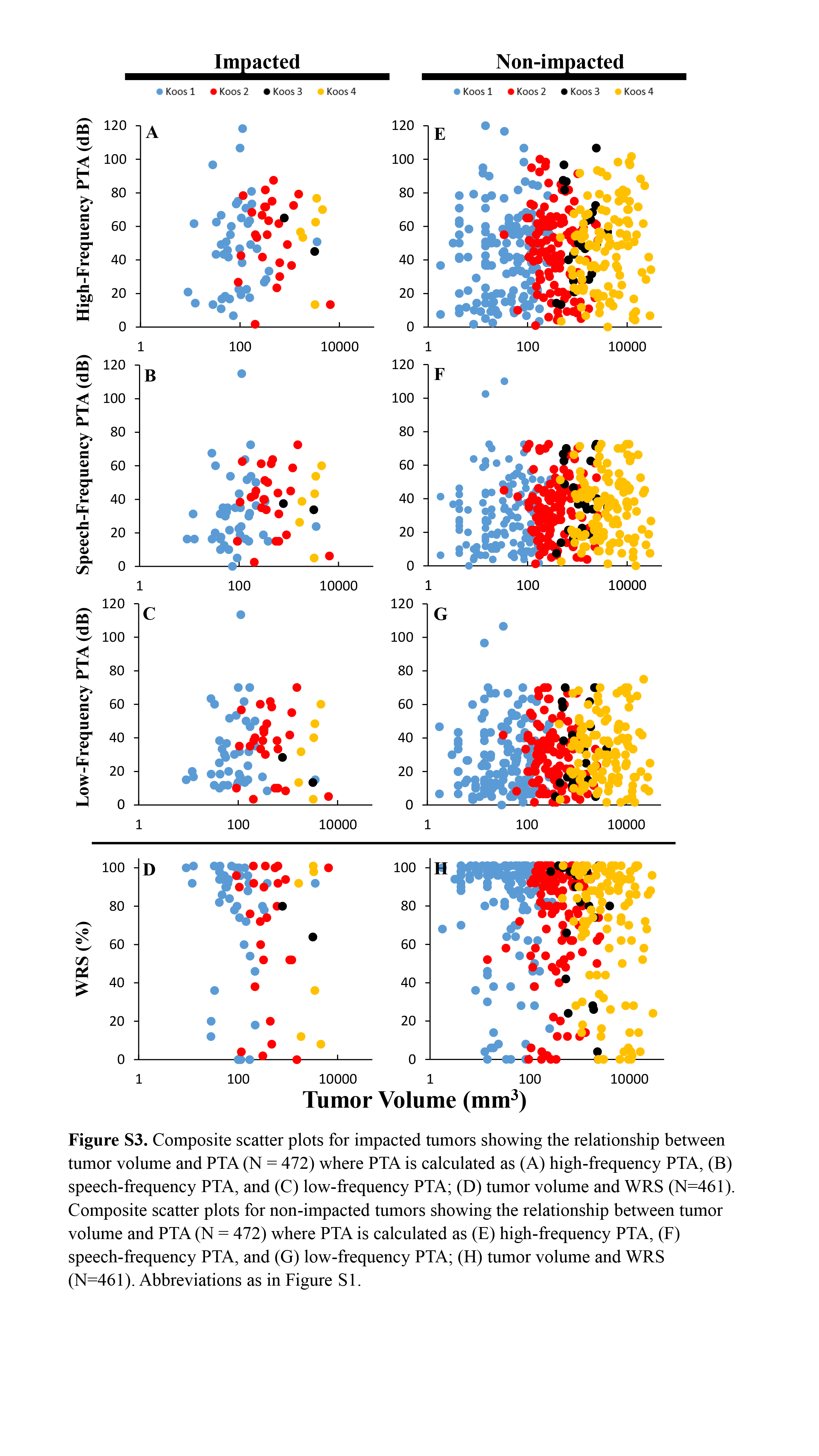

Supplement: Supplementary file 3 [file Image_3.tif]
